# The Development and Characteristics of Ancient Harbours—Applying the PADM Chart to the Case Studies of Ostia and Portus

**DOI:** 10.1371/journal.pone.0162587

**Published:** 2016-09-15

**Authors:** Ferreol Salomon, Simon Keay, Nicolas Carayon, Jean-Philippe Goiran

**Affiliations:** 1 Department of Archaeology, Faculty of Humanities, University of Southampton, Avenue Campus, Southampton SO17 1BF, Great Britain; 2 Centre National de la Recherche Scientifique (CNRS), UMR 5133-Archéorient, MOM, 7 rue Raulin, 69007 Lyon, France; New York State Museum, UNITED STATES

## Abstract

Over the last 20 years, the geoarchaeology of ancient harbours has been a very active area of research around the Mediterranean basin, generating much palaeoenvironmental data from many sites, including estimations of sedimentation rates, the height of the ancient sea-level at different dates and palaeo-geographical reconstructions. Combining this information has proved a major challenge. This article proposes a new chart called the *Palaeoenvironmental Age-Depth Model* (PADM chart), that allows the researchers to combine all relevant indicators in order to estimate harbour potential of a given ancient port, and to generate comparable data between harbours in terms of degree of closure and water depth available against a synchronised chronology. This new approach, developed in the context of the ERC-funded RoMP Portuslimen project, takes into account estimations of water depths relating to differing Roman ship draughts at different periods. It is tested against the palaeoenvironmental evidence published over 10 years from two Roman harbours located at the mouth of the river Tiber: Ostia and Portus. This reveals that: (1) there has been an underestimate of the real sedimentation rates due to the margins of error of the radiocarbon dates; (2) there was effective control of the water column by dredging; (3) there were different periods of control of the sedimentation. We suggest that the navigability of the Ostia harbour by ships with shallower draughts was maintained until sometime between the 2^nd^ c. BC and 1^st^ c. AD, while at Portus it was retained until the 6^th^—7^th^ c. AD.

## Introduction

Recent historical and archaeological research into harbours highlights the multiplicity of harbour types, as well as their synchronicity, diachronicity, and their hierarchies [1–3]. Many modalities for ships coming alongside a shoreline could coexist in a similar period and in the same harbour system. These were contingent upon the type of ship involved, the shoreline (rocky, sandy etc.) and its configuration (bay, lagoon, meandering river channel etc.), and any anthropic modifications (with/without structure, pontoon, moles etc.) that it may have undergone since antiquity.

Geoarchaeological studies, by contrast, have focused mainly upon enclosed harbour basins characterised by such artificial structures as quays and moles [4–6]. As such, specific and practical geoarchaeological concepts for dealing with this kind of archaeological context have been developed, i.e. “ancient harbour muds” included within an “ancient harbour para-sequence” [7]. Recently, geoarchaeological studies have adopted a different perspective concerning harbour environments [8,9], and have provided more complex typologies [10,11].

This article builds upon this earlier work, by incorporating the concepts of “navigability”, and “accessibility” as expressed in papers by Boetto [12] and Morhange et al. [11] and developing the concept of “harbour potential” in geoarchaeology. In particular, attention is directed towards issues related to the operability of selected harbours and their modes of use, taking into account relevant natural and topographical constraints. Drawing upon this more theoretical approach to the study of harbours, we propose the use of a new chart, called the *Palaeoenvironmental Age-Depth Model* (PADM). The PADM chart brings together stratigraphic data, palaeoenvironmental analysis, dating evidence and a hypothesized relative sea-level curve in a single age-depth model. The model allows the researcher to keep better control of the empirical data, such as the stratigraphic sequence of harbours, the analysis of deep cores and associated dating evidence, and to use it to evaluate harbour-use potential through time by means of bathymetric reconstructions.

**Fig 1:**
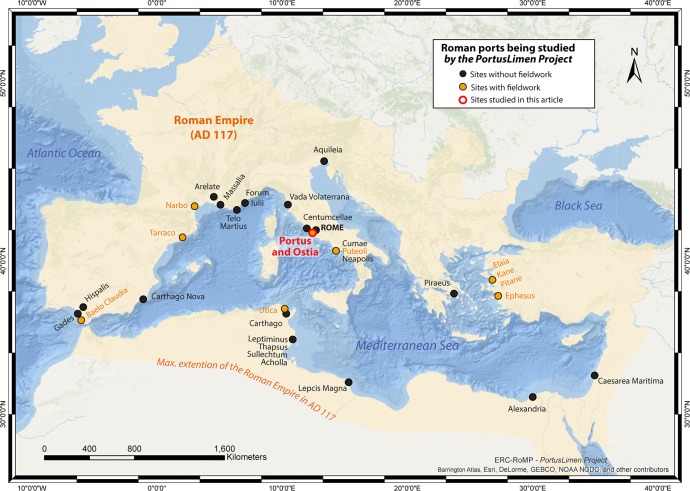
Roman ports being studied in the PortusLimen Project (ERC-RoMP) and the harbours discussed in this paper (Ostia and Portus).

**Fig 2:**
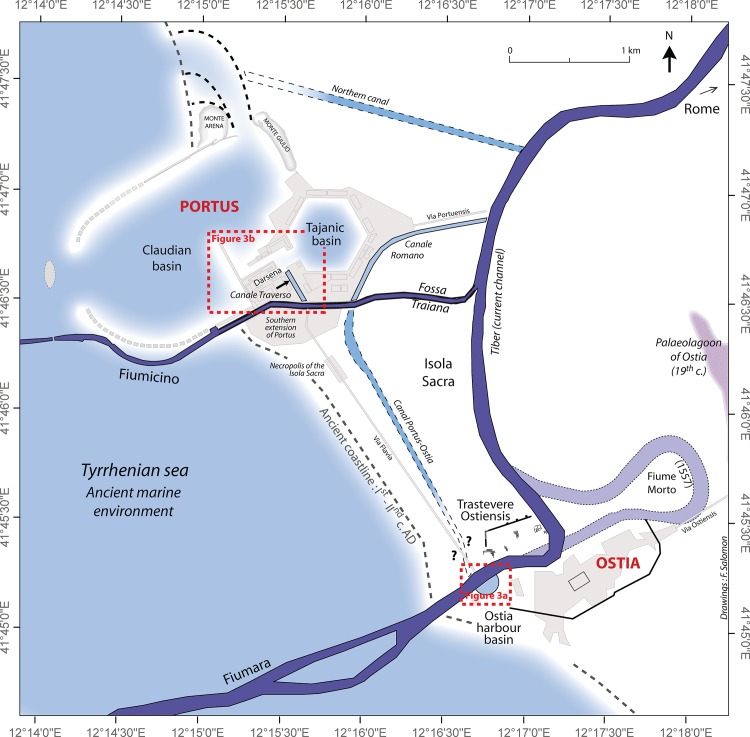
Ostia and Portus during the 2^nd^ c. AD. The map shows the location of the harbour basins, the canals that connected the two ports and their relationship to the Tiber.

The value of this new approach is explored by means of a comparative analysis of evidence from Roman harbour basin sediments from Ostia [13,14] and Portus [15–17] (Figs 1 and 2). We propose to bring together and to reinterpret the palaeoenvironmental data published from both sites over the past 10 years. It is argued that this approach sheds new light upon the operability and use through time of both basins and that, in particular, there was a correlation between dredging and ship draught.

## State of the Art

### Geoarchaeology of Ancient Harbours: toward the PADM chart

In geoarchaeology, a harbour is considered to be a geomorphological unit with inputs and outputs of water and sediments [4–6]. Vertically, the harbour sediments settle between two horizons, the katolimenic limit which marks the bottom of the deepest harbour, and can be either natural or anthropic, and the sea-level. Between these two limits there is a mesolimenic limit that fluctuates up or down, depending on the rate of sedimentation, erosion and dredging [15]. This approach has led to the development of two distinct scales of harbour analysis, based upon the *degree of artificialisation* and the *degree of protection* against the currents. The former applies to the sedimentary content, the rhythm of dredging and the importance of the infrastructure [10], while the latter is related to the concepts of “harbour mud” and “harbour parasequence” [7].

This concept has been usefully and effectively applied to many sites, notably Caesarea Maritima [18], Marseille [19], Alexandria [20], Tyre [21], Fréjus [22] and Portus [15] etc. The “ancient harbour muds” correspond to a “shift in the granularity” that “translate into the degree of harbour protection” [23]. These deposits offer data of a high potential from a geoscience perspective [4–6]. However, such sedimentary sequences are not continuous through time. Control of the high rate of sedimentation was, and still is, the major issue for the management of harbour basins. Basins were dredged in antiquity in order to minimise this threat to the utility of harbours. There is evidence from excavations for dredging activity at the bottom of the basins, such as for the ancient harbour of Naples [24], even though it is still difficult to identify them through sedimentary cores [25]. When dredging does take place, it creates gaps in the harbour sequences and effectively “reworks” sediment, that probably leads to several reversed chronologies in the stratigraphy [25].

Different kinds of research are revealing an increasingly complex range of ancient harbours [1,2,23,26,27]. Thus, for example, it is important to note that harbours are not only characterised by “harbour muds“. This is clearest at the harbour of Portus in the Tiber delta. By the second quarter of the 2^nd^ c AD it comprised three basins that operated together down to the Early Medieval period. Of its two major basins, the larger Claudian one is filled with sand, while the smaller Trajanic basin was filled with mud [15].

The *Palaeoenvironmental Age-Depth Model* (PADM) chart is a multi-parameter age-depth model bringing together data relative to heights/depths and dates that has been developed for the ERC funded RoMP/Portuslimen project (www.portuslimen.eu). It provides a new insight into the geoarchaeology of harbours by focusing on their water columns through time in addition to the degree to which they were protected and possibly provided with artificial infrastructure. Coastal environments are particularly well adapted for the use of this PADM chart, given that morphologies, palaeoenvironments and sedimentation are controlled by sea-level. The initial development of the PADM chart in the context of ancient harbours was first proposed by Salomon et al. [16].

### The Research Focus

By the second century AD, Imperial Rome was served by several ports that acted as nodes in what could be understood as a poly-focal hub or “port system”, comprising both banks of the Tiber within the City itself (particularly the area of the *Emporium*), Portus, Ostia, Centumcellae (Civitavecchia) and Puteoli (Pozzuoli), with Antium (Anzio) and Tarracina (Terracina) playing lesser roles. The term “system” is used here to loosely describe the close inter-relationships and connections between all seven ports. Within this port-system, Ostia and Portus were the two key sites that mediated Rome’s relationship with the rest of the Mediterranean. The former was a port city that was located on the banks of the Tiber and close to its mouth c. 35km west of the City, while the latter was a harbour complex that on the coast lay c. 3km to the north of Ostia. Both ports were connected to each other by a canal and road, while Portus was connected to the Tiber by two canals (Figs 1 and 2).

**Fig 3:**
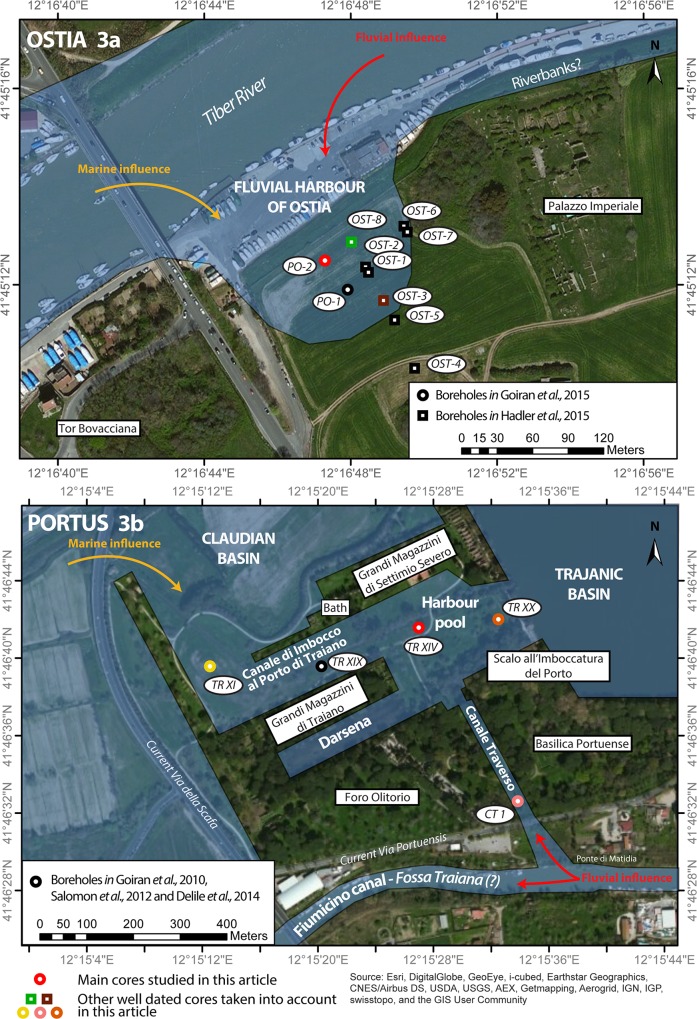
Borehole locations in the harbour basin of Ostia (upper) and at Portus (lower). Only the central harbour pool area of Portus with mixed fluvial and marine influences is taken into account here for Portus. The location of the cores with the best-dated stratigraphic sequences are visible on these two maps.

Ostia is much the better understood of the two ports in terms of its history, commercial organization, society and topography [28–30]. But far less is known about its harbour facilities and maritime façade. This has been remedied somewhat in recent years by the results of the geophysical survey carried out on the fluvial harbour of Ostia [14,31], and the cores drilled in the harbour [13,14] (Fig 3) and river mouth [32], as well as in the palaeomeander of the Tiber to the east of Ostia [33].

By contrast, the maritime infrastructure of Portus, has been the focus of much sustained research over the last decade, making it one of the most intensively studied Roman port sites in the Mediterranean. Many types of geophysical survey have been undertaken (magnetometry, Ground Penetrating Radar and Electrical Resistance Tomography) [34,35], followed by excavations in some areas [35], as well as surface clearance and topographical study [36,37]. Furthermore, at least five teams of geo-archaeologists have worked in the area [15,38–41], undertaking sedimentology, and analyses of macrofauna, microfauna, vegetal macro and micro-remains and geochemistry (Fig 3).

While our understanding of the harbour infrastructure of Ostia is clearly less advanced than that of Portus, there is now enough data to make a comparison possible. Taken together, they are ideal for the development of the inter-disciplinary PADM approach to the study of harbour infrastructure.

## Concepts and Methods for Building the PADM Charts of Harbour Contexts

Successive steps are taken in the construction of the *Palaeoenvironmental Age-Depth Model* (PADM) chart for one core drilled in the harbour of Ostia (PO-2) and for another drilled in the harbour of Portus (TR-XIV) (Figs 2 and 3). The choice of these two cores was driven by the amount of the radiocarbon dating evidence available and the diversity of the palaeoenvironmental analysis undertaken on their stratigraphic sequences. Preparatory steps included (1) the fieldwork and the drilling/excavation, the analysis in laboratory with (2) palaeoenvironmental analysis and (3) dates, and (4) the construction of the PADM chart.

The PADM is drawn on a classic age-depth model. However, the stratigraphic sequence is drawn on the vertical axis (Y-axis) of the PADM together with the significance of the palaeoenvironmental data analysed. The grain-size indicator of the hydrodynamism and the palaeo-ecological context are expressed together with the palaeoenvironmental context (lagoon, fluvial, or coastal). On the horizontal axis of the PADM are transposed the stratigraphic layers through the reconstructed sedimentation curve. The interpretations of the harbour potential / operability relate to this axis. The PADM is completed by additional chronological information relevant to the core and the harbour considered, and by a reconstructed relative local sea-level curve. The space between the reconstructed sedimentation and the relative sea-level curves correspond to the water column available for navigation through time.

### Palaeoenvironmental analysis

After the conclusion of the excavations and coring, sediments are removed from the site and studied in the laboratory—all necessary permits were obtained for this study, which complied with all relevant regulations; *Soprintendenza Speciale per il Colosseo*, *Museo Nazionale Romano e Area Archeologica di Roma (Ufficio di Ostia*) authorized the access to the drilled fields. Palaeoenvironmental data from Ostia and Portus synthesised in this article is available in several published articles [13–17]. Non-destructive analyses are performed to characterize the stratigraphic sequences sampled (magnetic susceptibility, itrax scanner etc.).

Destructive analysis starts with sampling the sedimentary units. This will differ according to the analyses needed, ranging from high to low resolution. Sedimentological analysis includes wet and dry sieving in order to identify the texture of sediments. More precise information about grain-size distribution can be obtained by using laser grain sizers. These results provide important information about hydrodynamism (Portus: [15]; Ostia: [13,14]). Bio-indicators are an important aid to identifying biota, and estimating the freshwater/marine water balance: shells, ostracods (Portus: [15,42]; Ostia: [13,43]), vegetational macroremains (Portus: [44]) and pollen (Portus: [41]; Ostia: [43]).

The construction of the PADM chart is based upon the core sequence subdivision identified by different authors (log, stratigraphy). When possible, the diagram of the texture and ostracods are included next to the log. Description of the units includes the main grain size characteristics derived from the different component materials (clay, silts, sand, gravels, muddy etc.) and the salinity contexts (freshwater/brackish/marine). The presence of *Posidonia*, shells, ceramics, and organic layers are also recorded on the stratigraphic logs. References to the different studies are of course recorded on the graphs.

### Chronology

The first level of chronological information is derived from the stratigraphic sequence itself, with the succession of the different deposits providing a solid relative framework. This makes it possible for the researcher to critique and discuss the absolute dating undertaken on the core sequence. This “stratigraphic control” is one of the strengths of the PADM chart, in which the stratigraphy provides the framework of a classic age-depth model.

A quick glance at the geoarchaeological literature relating to ancient harbours underlines the importance of radiocarbon dates in the construction of absolute chronologies. The stratigraphic sequences of Ostia and Portus are mainly dated by this means, sometimes taking into account evidence from ceramics as well. We re-calibrated all radiocarbon date by using the software OxCal (https://c14.arch.ox.ac.uk/oxcal/OxCal.html); radiocarbon dates using the marine calibration curve (Marine13) are represented on the figures in blue, while dates calibrated with the continental curve (IntCal13) are shown in red (S1 Table) [45]. However, we chose not to generate an age-depth curve mathematically, since harbour sequences can be transformed by anthropic layers, dredging actvities etc.

### Sea-level indicators

Ostia and Portus encompass two separate harbour systems whose use was conditioned by the height of the sea-level. This means that determination of ancient sea-level indicators is fundamental to the construction of a PADM chart. A biological mean sea-level for the Tiber delta has been estimated on the basis of the presence of barnacles on the northern quay of the Claudian basin at Portus at a point c. 80cm below the present sea-level and dated to 2115 ± 30 BP, 230 to 450 AD (Code LY-4198 [46]). The local relative sea-level curve for the Tiber delta is reconstructed on the PADM charts using the present-day and the biological mean sea-levels described previously. This reconstructed relative sea-level curve might have integrated subsidence and uplift trends in the area of Portus over the last 1500–2000 years.

### Ships and use of the harbour space through time

Application of the PADM chart to the sedimentary sequence of the harbours makes it possible to focus upon their potential as navigable spaces through time. The height of the water column at a particular moment in time can be set against the estimated draught of a fully laden ancient ship or boat. Since there is a proportional relationship between this and the estimated size of ancient ships or boats, it is possible to gain an idea of the relative size of the ships that were able to use these harbours (Boetto 2010: Tableau 1 [12]).

Ships that comprised the Alexandrian grain fleet, which were supposed be amongst the largest in the Roman Mediterranean [47], would have been able to penetrate the harbour of Portus [12,15,47,48,49]. One related question that the PADM might be able to help us answer would be for how long this held true? Another might be whether large ships like this were able to use all of the basins in the port, or if they were restricted to certain water spaces, with others being used by smaller ships and boats? The PADM chart might also be able to help us learn whether the harbour of Ostia was designed to hold similarly large ships. One could also ask which parts of the harbours of Portus and Ostia were best suited to the shallower draught Tiber river boats, the *naves* c*audicariae*, and whether there was a time when this also became difficult? Might the PADM chart provide us with evidence that would suggest that there were attempts at dredging, as in other parts of the Roman Mediterranean, and if so, whether it was being undertaken with a view to enabling the harbour to accommodate boats of a particular draught, and thus size?

## Developing the Case Studies of Ostia and Portus

### The Republican and early Imperial river harbour of Ostia

**Fig 4:**
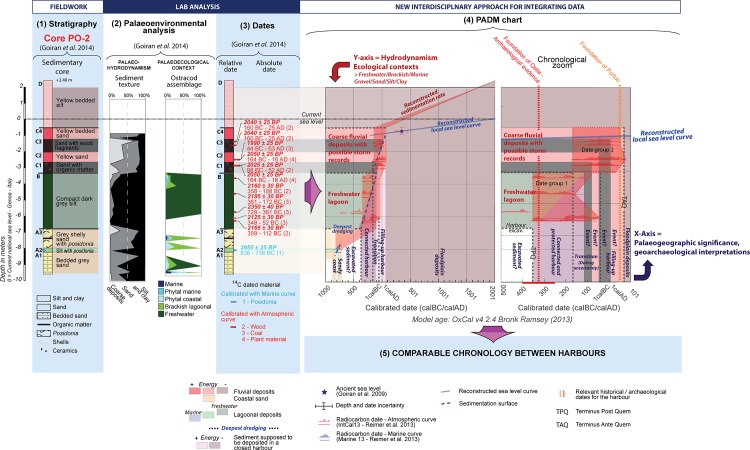
PADM of the core PO-2 drilled in the harbour of Ostia. This figure presents the analytical stages in the development of the Palaeoenvironmental Age-Depth Model (PADM chart) for core PO-2: (1) stratigraphy of four main units including pre-harbour deposits (Unit A), deposits in the harbour (Units B and C); post-harbour deposits (Unit C and D); (2) results of the palaeoenvironmental analysis (sediment texture and palaeoecological context of the ostracod assemblages); (3) stratigraphy and radiocarbon dates; (4) PADM. The PADM proposes an integrated age-depth model that includes stratigraphic and palaeoenvironmental data (hydrodynamism and ecological contexts on the Y-axis), their interpretation in terms of palaeogeography or geoarchaeological significance in a harbour context (X-axis), a reconstructed sedimentation curve, and a reconstructed relative sea-level curve. Core PO-2 located in the middle of the harbour of Ostia, was the most representative and provided the most complete dated sequence. The core sequence PO-2 mainly records sedimentation between the 4^th^ century BC and the 1^st^ century AD.

Core PO-2 was drilled in the middle of the harbour basin at Ostia, which is situated on the southern side of the Tiber channel (Fig 3). It was studied by means of a wide range of palaeoenvironmental analyses (grain-size, C/M Passega diagram, ostracods, and pollen) and has provided the greatest number of key radiocarbon dates from a single sequence at the port. Full descriptions of the core and the analyses can be found in [13,50]. Fig 4 synthesises its stratigraphic sequence together with a textural diagram (coarse deposits, sands, silts/clays), an ostracod diagram and the twelve available radiocarbon dates. All of these analyses have been brought together in an age-depth model and, together with the reconstructed sea-level curve, provide us with the interpretative PADM chart of the core PO-2. Uncertainty over the chronology and the rhythm of dredging activity has meant that only very basic sedimentation curves have been produced for each stratigraphic unit; they take into account the full thicknesses and time spans of the radiocarbon dates of each single sedimentary unit.

The sequence in PO-2 can be subdivided into four stratigraphic units. Unit A was composed mainly of very well sorted sand that correspond to fluvio-coastal sediments deposited during a progradational phase of the Tiber river mouth during the first part of the 1^st^ millennium BC (Date code: Ly-8066 –S1 Table). Unit B above it reveals a totally different environment composed of dark grey silts.

The stratigraphic discontinuity between Units A and B has been interpreted as the bottom of the fluvial harbour basin excavated at Ostia [13,14]. The sediments in Unit B are typical of the “harbour muds” found in enclosed harbour basins [4,5]. In a ‘bottom-up’ reading of the stratigraphy, we can observe a gap in the chronology between Unit A dated to 836–736 BC (Date code: Ly-8066) and the first date of the Unit B at 359–112 BC (Date code: Ly-9092). All radiocarbon dates in Unit B fall within the range between the 4^th^ and 2^nd^ c. BC and suggest that there was a chronological gap between this and the Unit A below. One date recorded in Unit B, between 729 and 361 BC (Date code: Ly-9094), could in theory fill this gap. However, this older date cannot be taken into account, since the presence of two more recent dates below it implies a *terminus post quem* succession starting in 359 BC at the bottom of the Unit B (Date code: Ly-9092), moving to 348 BC for the second date (Date code: Ly-9093). The chronostratigraphic gap between Unit A and Unit B, at 6m below the reconstructed sea-level of the 4^th^–3^rd^ c. BC, was almost certainly caused by the excavation of the harbour basin of Ostia, or at least deeper dredging within it, and consequently suggests that the harbour had anthropic origins.

The palaeoenvironmental analyses undertaken on the core sediments suggest that the Ostian “harbour mud” in Unit B corresponds to freshwater lagoon deposits created through the influence of marine water. This unit is overlapped by several layers of coarse sediments deriving from one or several high energy events dated between 164 BC and AD 63 AD (Unit C) (Date codes: Ly-8064, Ly-8063, Ly-8062, Ly-8061, Ly-8060, Ly-8059); these are to be interpreted as coarse fluvial sediments transported by floods [13] with possible high energy deposits coming from the sea [14]. The sequence is eventually covered by silty floodplain deposits (Unit D).

**Fig 5:**
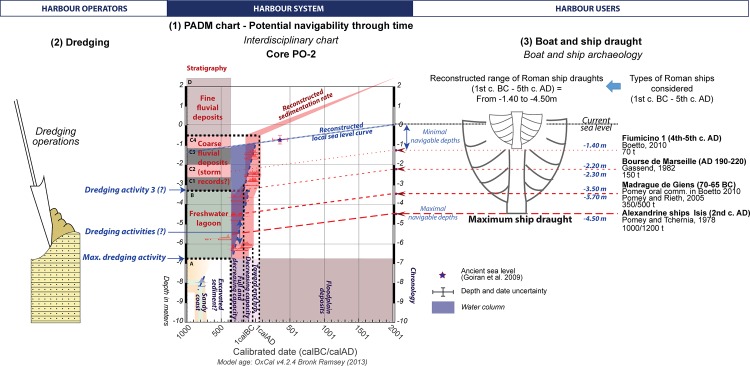
Harbour operation and PADM of the core PO-2. PADM chart based upon core PO-2 that represents how the harbour basin of Ostia may have been used. The chart incorporates stratigraphic data as well as factoring in the dredging level hypothesis and possible ship draughts. The PADM shows different stages of harbour potential through time and proposes different hypotheses relating to levels of dredging.

Fig 5 provides a PADM chart specifically applied to a harbour context and presents a new set of interpretations. Dredging is taken into account in regards to the stratigraphy and the radiocarbon dates. On the right hand side of Fig 5, these indicators are viewed in relation to the reconstructed draught of fully laden Roman ships [12]. The age-depth model of the harbour sequence at Ostia makes it possible to identify two chronological groups, one related to Unit B (4^th^–2^nd^ c. BC) and a second related to Unit C (2^nd^ c. BC to 1^st^ c. AD). The chronological spans of each of these overlaps within each unit but also between the two units for the end of the 2^nd^ c.–1^st^ c. BC. These overlaps are linked to the calibration curve for the radiocarbon dates. In this context it is impossible to observe age inversions or temporal gaps from the bottom of the harbour basin.

The discontinuity between Units B and C is a trapped level of a harbour in siltation which could only have been used by fully laden ships with a draught of less than 2.20 / 2.30 m. When the flood events in Unit C took place, the harbour had lost its initial maximum capacity in terms of depth. Afterward, the harbour basin was possibly maintained for smaller ships or boats between Sub-Units C1 / C2 and C2 / C3, and was then filled with Units C3 and C4, with a *terminus ante quem* of AD 63 (Date code: Ly-8061).

**Fig 6:**
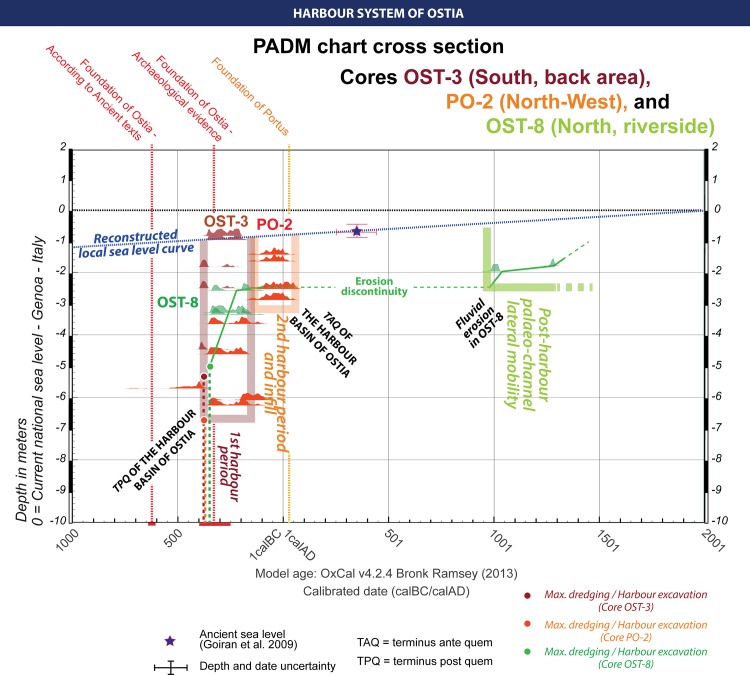
PADM cross section through the central sector of the river harbour of Ostia. It draws together the dating sequences of PO-2 and OST-8 (central area) and OST-3 (south side). The three periods and three areas of the harbour follow Goiran et al., 2014 and Hadler et al., 2015.

The interpretative PADM chart provides a useful tool for visualising the evolution of the bathymetric or water column of the Ostia harbour basin through time. In particular, the chronostratigraphic evidence from Core PO-2 discussed above is compared to other sequences and age models derived from cores drilled towards the centre of the basin and data published by Goiran *et al*. and Hadler *et al*. [13,14] (Fig 6). Core OST-3 was derived from a borehole that lay c. 60m to the south-east of borehole PO-2 while core OST-8 was derived from a borehole that lay c. 30m to its north-east (Fig 3 and S1–S3 Figs for detailed PADM charts) [14].

Taken together, the three core sequences confirm the idea of a first harbour phase dating to between the 4^th^ and the 2^nd^ c. BC (c. 400 and 125 BC). The only core sequence to provide dating evidence for sediment deposited in the 1^st^ c BC / 1^st^ c. AD was OST-5, but this seems to lie outside the harbour basin toward the south. The C-14 analyses provide dates of 1999 ± 19 BP, 43 BC-52 AD (Code: MAMS 19755) and 1930 ± 18, 27–125 AD (Code: MAMS 19756) at 0.82 and 0.22 above sea-level. Archaeological dating of ceramic fragments from Cores OST-1 and OST-3 drilled in the harbour basin provide a date range of between 40 BC and AD 150 from a maximum depth of c. 1.75 below the present sea-level, or less than 1m below the reconstructed sea-level of that period (Cores OST-1, OST-3 and OST-5 [14]).

Radiocarbon dates taken from points between c. -2 and 0m below current sea-level in OST-2 and 8 indicate dates of between 10^th^ and 13^th^ c. AD (Codes: MAMS 19747, 19763, 19764), and are related to the erosion of the Tiber riverbank during the Medieval period, and the removal of the upper part of the Roman harbour sequence [14]. The PADM chart in Fig 6 provides clear evidence for the stratigraphic discontinuity caused by the lateral channel erosion in Core OST-8 during the medieval period (Fig 6 and S1 Fig). Spatially, the maximum extent of erosion seems to reach a line between Cores OST-8/OST-2 and Cores PO-2/OST-1. However, radiocarbon and archaeological dates suggest that the harbour of Ostia is maintained until the 1^st^ c. AD (Cores PO-1, PO-2, OST-1), but with reduced depth and, thus, capacity. In fact, the water column is less than c. 1m below the reconstructed sea-level of that period and the harbour was no longer well-protected against coarse flood deposits. Consequently, the hypothesis proposed by Hadler et al. [14] suggesting the “subsequent establishment” of a fluvial harbour in the “1^st^ c AD onwards” based on the data from Cores OST-1, OST-2 and OST-8, does not seem to match the palaeoenvironmental evidence.

Central to any consideration of the functionality of the harbour basin at Ostia is the accessibility of the harbour from the Tiber and the navigability of the river mouth [33]. River mouths are particularly dynamic sedimentary environments, and the formation of sandbanks at the mouth of the river was clearly a major obstacle [32]. Livy (*Ab Urbe Condita*, 29, 14 [51]) recounts how a ship bearing the Magna Mater ran aground on a sandbank at the mouth of the Tiber in 205–204 BC, while Strabo (*Geography*, 5, 3, 5 [52]), who was writing in the 1^st^ c AD, described Ostia as *alímenos*, or without a sheltered harbour. He goes on to say that since fully laden larger ships had difficulty sailing past the sandbank at the mouth of the Tiber, they had to offload cargoes on to smaller ships to enable them to move up the river into Ostia. At the same time, one should be cautious in assuming that what may have been temporary difficulties were permanent constraints. Nevertheless, this information points to the challenges inherent in the larger sea-going ships using the harbour, and may be an argument in favour of it being used most heavily by lighters serving larger ships that were moored offshore, and river boats that would have carried cargoes upriver to Rome.

### Early to late Imperial Portus: a maritime harbour with mixed influences

**Fig 7:**
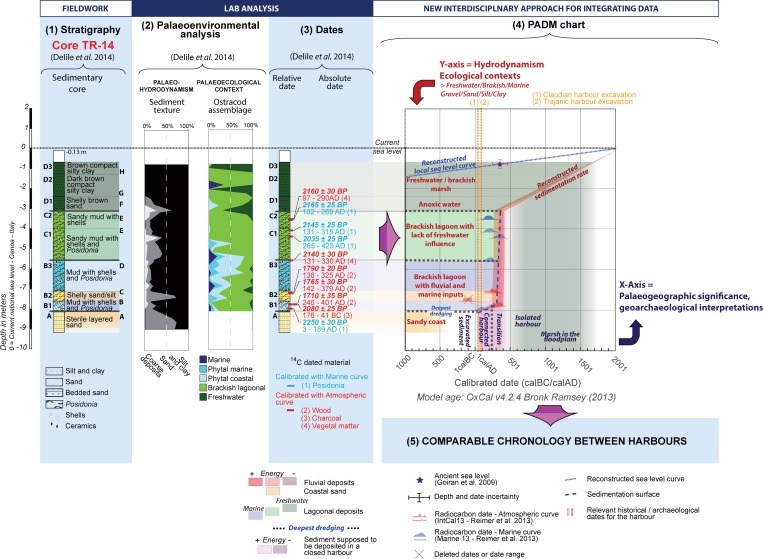
PADM of the core TR-14 drilled in the harbour of Portus. This figure represent the stages in the development of the PADM chart for core TR-XIV, the most complete dated sequence from Portus. Located at the junction of fluvial and marine influences within the port, core TR-XIV is representative of the water dynamics between the Claudian and Trajanic basins and the *Fossa Traiana*. The core sequence TR-14 mainly records sedimentation between the 2^nd^ and the 4^th^ century AD.

**Fig 8:**
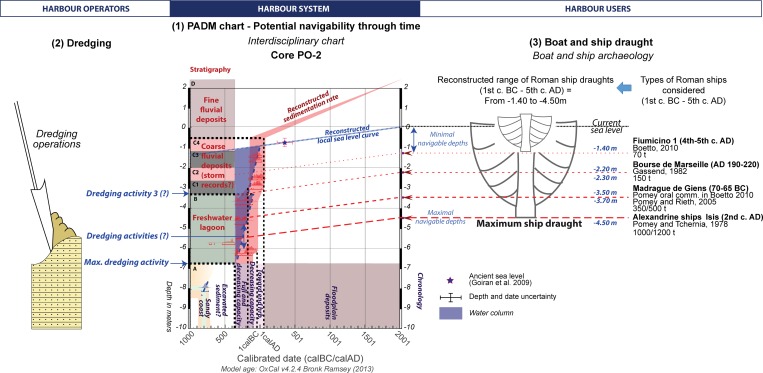
Harbour operation and PADM of the core TR-14. **T**his figure shows the PADM chart of the operating life of the pool of the harbours at Portus as reflected in the sedimentary sequence of core TR-XIV, and expressed by four stratigraphic units. The PADM chart incorporates stratigraphic data as well as factoring in the dredging level hypothesis and possible ship draughts. Deep dredging activities may have occurred in the 3^rd^ -4^th^ c. AD.

Core TR-XIV was taken from a borehole drilled in what had been the pool of the harbour (Fig 3), to the west of the Trajanic basin. This central position was originally open to both marine and fluvial influences, with the former arriving from the Claudian basin to the north-west by means of the *Canale di Imbocco al Porto di Traiano*, and the latter from the Tiber and *Fossa Traiana* by means of the *Canale Trasverso* to the south. Many palaeoenvironmental analyses (grain-size, C/M Passega diagram, ostracods, and geochemical data) and ten radiocarbon dates (Detailed analyses in Salomon et al., 2012 [16] and Delile et al., 2014 [17]) provide the best dated palaeoenvironmental record of the sedimentary sequences at Portus. Fig 7 shows the full stratigraphic log and record of the texture, ostracod assemblage and dates. The PADM charts in Figs 7 and 8 combine evidence for the palaeoenvironment, dates, reconstructed sedimentation rate, reconstructed sea-level curve, and two relevant historical events for the harbour.

Unit A corresponds to a laminated sandy sediment, a facies that is related to coastal deposits and most probably pre-dates the establishment of Portus in the mid 1^st^ c AD [15–17]. A sharp change in the character of sedimentation occurs at c. 8m below current sea-level, between Units A and B. Sub-Unit B1 is a muddy deposit from a brackish lagoonal environment that exhibits marine influences. It is dated to between AD 3 and 189 (Code: Lyon-8776) and must relate to activity within the closed harbour of Portus [4,5]. Unlike Ostia, however, there is no chronological gap in the sequence of TR-XIV that would signal the excavation of the pool of the harbour. However, the dates of the excavation of the Claudian and Trajanic basins are quite well defined. The former must have been completed by c. AD 46 (Keay et al. 2005: 297–305 [34]), and the latter by c. AD 112–114 (Keay et al. 2012: 504 [53]), and both of these dates provide a useful *terminus post quem* for each basin. Since core TR-XIV was located towards the centre of the pool that lies between the Claudian and Trajanic basins, its stratigraphic sequence could be either related to either of the harbours. Consequently, the earliest deposits preserved in the sequence can be dated to some time between AD 46 and 189, an adjusted date that takes into account the margins of error inherent in the *terminus post quem* for the establishment of Portus and the first radiocarbon date.

The harbour sequence from the bottom of Unit B to the top of Unit C shows the isolation of the pool from both regular freshwater fluvial influences and those from a maritime environment. Unit B corresponds to well-oxygenated brackish lagoonal muds with strong marine inputs and freshwater supply (Units B1 and B3) interrupted by a layer of sands that were probably deposited during a flood (Unit B2) [17]. Unit C1 exhibits a growing influence of seawater, and subsequently the effects of the formation of what amounts to a closed lagoon, a development that has been interpreted in terms of the closure of the *Canale Trasverso* [17]. Geochemical analysis and a brackish ostracod assemblage in Unit C2 provide evidence for oxygen deficiency, or anoxia. This indicates that the pool was isolated from the river as well as from the sea, and reaches its climax in Unit C3. Finally, the sediments corresponding to the latest units, D1 and D2, were deposited in a brackish to freshwater lagoonal environment with inputs of terrigenous particles of silty clay dispersed by Tiber floods [17].

The PADM in Figs 7 and 8 conceptualizes the usage through time of the harbour pool at Portus in terms of four stratigraphic units of differing environment and depth. This makes it possible to see how the pool would have been initially able to sustain the use of larger ships, and how they would have given way to ships and boats of lesser draught through time.

Establishing a chronological framework for this development however is not without its challenges. Three dates were recorded in the Sub-Unit B1 and they illustrate the importance of using several dates to establish the chronology of a single layer. As we have already seen, the first radiocarbon date of AD 3 to 189 (Lyon 8776) at a depth of 8.03m b.s.l. is compatible with the establishment of the broad infrastructure of both the Claudian and Trajanic harbour basins. However, a charcoal fragment at the slightly higher level of 7.82m b.s.l. has yielded a date of 176–41 BC (Code: Lyon-8877) that precedes the establishment of the port. Chronological inversion of this kind may be suggestive of dredging activity [25]. Furthermore, a piece of wood from the same level was dated to AD 246–401 (Code Lyon-8876); the gap that exists between the latter date and the deeper date of AD 3–189 further reinforces the argument that the sequence of sediments in sub-Unit B1 may have been affected by dredging (no deposition at all for several centuries is possible but seems impossible in the strong sedimentary dynamics of the Tiber delta). In any event, *per se* the date of AD 246 from the wood fragment provides a *terminus post quem* for the subsequent sedimentary deposits. When considered together, therefore, the dating evidence from the sediments in core TR-XIV point to a gap in the depositional sequence between the 1^st^ and mid 3^rd^ c AD, which can presumably be interpreted as a period during which the pool could have been maintained by one or more dredging horizons and possibly frequented by ships with larger draught. The subsequent quick sedimentation, by contrast, may be indicative of different conditions of sedimentation, shallower dredging episodes and the use of the pool by boats or ships of shallower draught.

**Fig 9:**
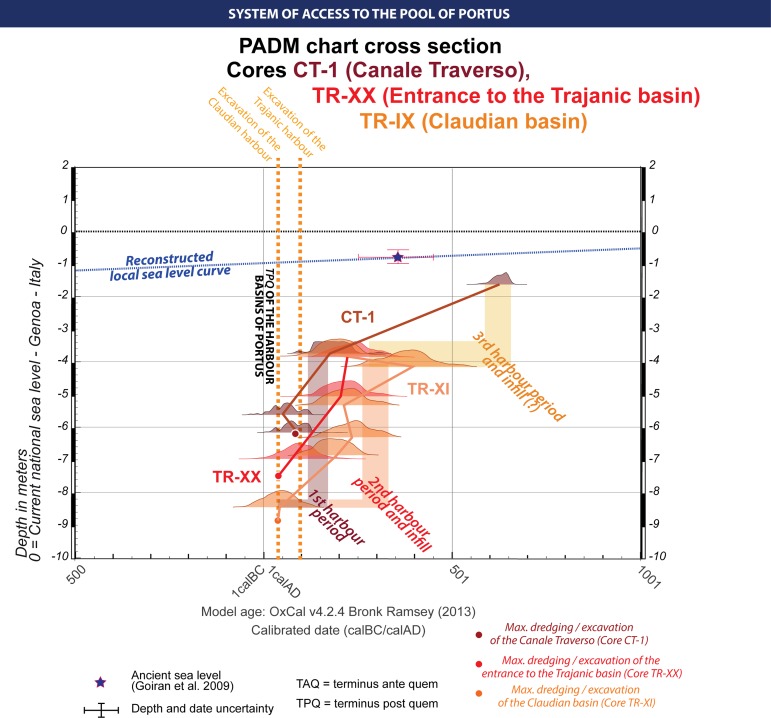
PADM of the accesses around the harbour pool of Portus. It draws together the dating sequences of CT-1 (Canale Trasverso), TR-XX (Entrance to the Trajanic harbour basin) and TR-IX (Canale di Imbocco al Porto di Traiano, Claudian side) [15–17,56]. This figure suggests deep dredging activity during the 1^st^ to the 2^nd^ c. AD, a continuation of deep dredging and an infilling of the harbour between the 2^nd^ and the 4^th^ c. AD, and a harbour that was possibly maintained for smaller ships from the 4^th^ to the 7^th^ c. AD.

Evaluating the accessibility to the harbour pool from adjacent water spaces can be gauged indirectly from the analysis of a single core. This was achieved by Delile et al. (2014) [17] who was using geochemistry in order to characterize fluvial and marine influences. Alternatively, evaluating the accessibility can be undertaken from the analysis of several cores drilled in the different waterways in the harbour. Understanding the access to the harbour pool depends on the cores drilled in the *Canale Trasverso* (CT-1) and the *Canale di Imbocco al Porto di Traiano* (TR-XIX, TR-XI), which provide further information about the conditions and modalities of closure or maintenance. Fig 9 provides an integrated view of the age-depth models of TR-XX, TR-IX and CT-1. In a manner similar to TR-XIV, it is interesting to observe two period of activity of the harbour in TR-XX and TR-XI, a maintained deep harbour (1^st^–2^nd^ c. AD) and a silting harbour (3^rd^–5^th^ c. AD). For Core CT-1, the deep dredging observed in Cores TR-XX, TR-XI, TR-XIX or TR-XIV never happened, but the *Canale Traverso* is maintained for a c. 2m water column. A chronological gap between early 2^nd^ c. AD and AD 585 to 663 (Code: Lyon 6869), reveals possible episodes of dredging [16]. The absence of organic material made it impossible to date the final closure of the pool of the harbour, and it can only be suggested that the water column of the *Canale Traverso* in the 6^th^-7^th^ c. AD lay at c. 1m below the sea-level of that period (S2 and S3 Figs).

Looking a bit further afield, the access to the *Canale di Imbocco al Porto di Traiano* relates to the northern and western entrances to the Claudian basin [40,54,55].

## Discussion

This paper is the first step in the development of the concept of “harbour potential” in geoarchaeology by means of the *Palaeoenvironmental Age-Depth Model* (PADM). It takes into account the palaeoenvironmental conditions (hydrodynamism, marine/freshwater balance) and the available water column at different periods through time. The PADM is a graphical tool that standardizes and integrates stratigraphic data, palaeoenvironmental analysis, dating evidence and a hypothesized relative sea-level curve, in order to characterize and compare different coastal harbour regimes and their suitability for ships and boats of different sizes.

**Fig 10:**
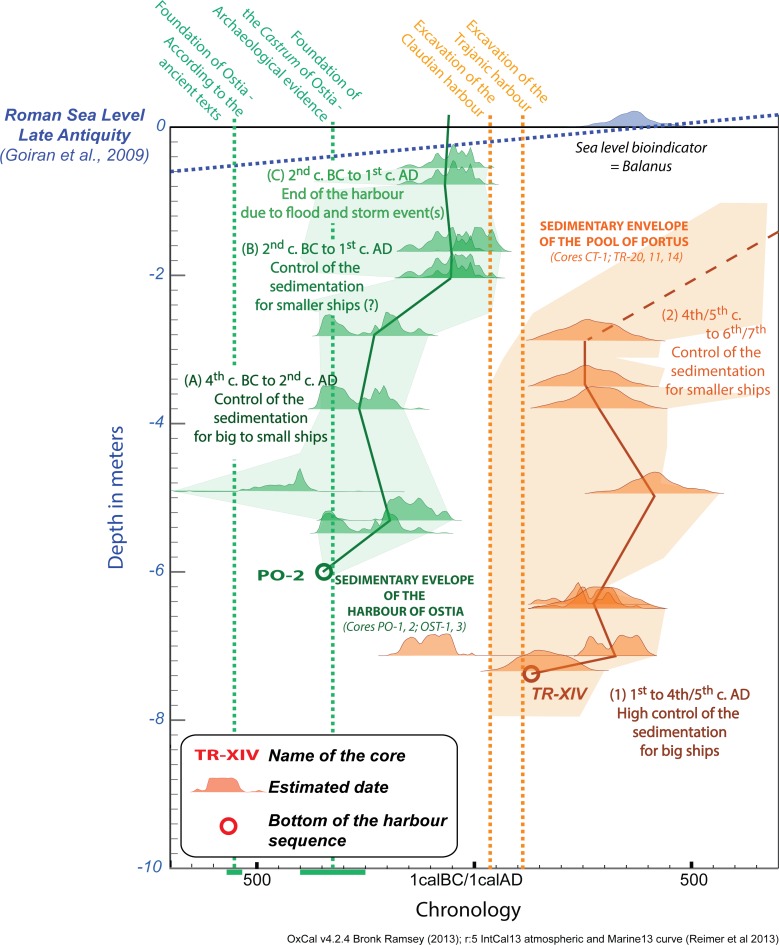
Sedimentary chrono-envelopes of the harbour sequences of Ostia and the harbour pool of Portus. The Chrono-envelopes are based on all the dating evidence from deposits related to defined harbour areas. Dating of the core PO-2 (Ostia) and TR-XIV (Pool of Portus) have been reported as part of their respective chrono-envelope. A clear change in harbour potential is evident in the chronological shift of harbour basin at the Tiber mouth, from Ostia to Portus in the 1^st^ c. AD.

The harbour at Ostia and the pool of the harbour complex at Portus were chosen as the case studies for this paper. Both were relatively close to one another within the Tiber delta, with similar sandy coast pre-portuary deposits, while the silting up of the Ostia harbour with fluvial deposits seems to have been almost complete by the time that the harbour pool, *Canale Trasverso* and Claudian basin at Portus begin to be used in the early 1^st^ c AD (Fig 10). Portus began to gradually silt up with possibly shallower dredging control from the 3^rd^ c AD onwards, although archaeological and textual evidence show that the port continued to function in some form until at least the 9^th^ c. AD [57]. The two chrono-envelopes of Ostia and Portus synthesise the dating evidence from the harbour of Ostia and the harbour pool at Portus (Fig 10), and make it possible to illustrate a comparison of the sedimentation in the two harbour through time.

PADM analyses in Figs 4 and 7 have made it possible to raise questions about the main characteristics of the deposits and the chronologies of the harbour at Ostia and the harbour pool at Portus. These relate to the function of ports and are defined here as navigability, accessibility, harbour potential and harbour operation. The analytical potential of the concept of navigability is based on estimating the depth of sedimentation in harbour basins in relation to the estimated sea-level at specific periods. Accessibility, by contrast, can be inferred from cores taken from several boreholes drilled at different key points in the port in order to evaluate the accessibility of water column for ships, or in an attempt to locate harbour entrances of the harbours [15,40] (Figs 6 and 9).

A second issue raised by the PADM charts concerns harbour operability, and in particular, the challenges inherent in evaluating the type and the quality of Roman harbours, their maintenance, their purpose and synchronicity with the evidence from other ports. Observed changes in the height of the water column at both harbours at different points in time also provides an important index of the maximum depth available for ship draught in different parts of the harbours (Figs 6, 9 and 10) [12,46]. This provides us with an important clue as to the scale of the ships and boats that were able to frequent the harbours. It suggests that in the case of Ostia, large fully-laden ships with a draught of up to 4.5m could have initially entered it from the 4^th^ c BC–2^nd^ c. BC. But gradual sediment build-up soon began to restrict clearance of the harbour bottom, despite episodes of dredging, down until the c. late 1^st^ BC/early 1^st^ c AD, when the water column was restricted to c. 1m –a depth that would rendered the harbour largely useless to large ships. At Portus, by contrast, large fully-laden ships with a draught of up to 4.5m could have passed through the harbour pool until the 3^rd^-5^th^ c. AD, after which time the sediment build up would have restricted passage to progressively smaller ships and boats.

**Fig 11:**
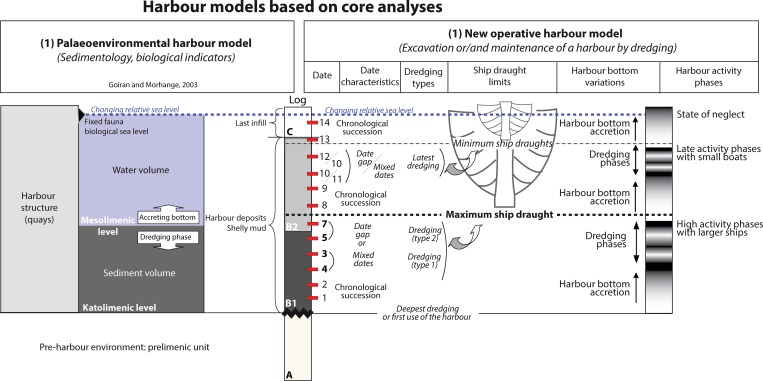
Model of an operating harbour based on the PADM analyses of the harbours of Ostia and Portus. The left side of the figure shows the harbour from a palaeoenvironmental perspective [4,5]. The right side shows the harbour sedimentation in a new interdisciplinary perspective, including data from ancient ship and boat reconstructions and a more detailed consideration of the dredging phases. This model suggests that as soon as a water column here was available, there was potential for navigation and the possibility of that the harbour continued to operate. However, the correlation of different core sequences is needed for a reconstruction of the accessibility of the harbour and a general understanding of its potential through time. The PADM makes diachronic perspectives of this model possible.

It is also noticeable that at both harbours there are discontinuities or chronological inversions in the sedimentary record that would seem to be best explained in terms of dredging horizons (Figs 5 and 8). Since these represented attempts at increasing the depth of the water column, it raises questions about the maintenance of the harbours, and the kinds of adaptation that were employed in the face of sedimentation and erosion (Fig 11). While control of the sediment budget depended upon *preventive actions* during the initial planning and layout of the harbour, including the careful siting of the quays, canals, locks and other infrastructure, short term actions such as dredging, combined with *adaptive actions*, such as the construction of new structures through time, were key to maintaining the operability of the harbour [16]. The minimum mean sedimentation rate for the period 4^th^ c. BC to 1^st^ c. AD in the Ostia basin would be c. 8.5 to 10.5 mm/yr (core PO-2). At Portus, by contrast, it has been calculated at 26.5 mm/yr for the period between AD 250 to 400 (core TR-XIV). A high sedimentation rate thus characterises the harbour basins of both Ostia and Portus during these two periods, although they are in fact constraints created by margins of error in the radiocarbon dates. The sedimentation rate during the periods tested could have been lower but it would have been correlatively much quicker in the same time span. The average sedimentation rate is thereby under-estimated when compared to the likely real maximum sedimentation rate.

Last but not least, a final issue raised by the PADM charts is that the kinds of analysis involved in their creation can be a useful measure for gauging the suitability of accessible navigable areas close to shore as potential ancient harbour sites. We focused in this paper on built enclosed harbours, but other kinds of port environments can be evaluated from the perspective of harbour potential: (1) water column availability, (2) accessibility and (3) palaeoenvironmental conditions / degree of closure. For example, it was recently demonstrated that the depth of the palaeo-lagoon at Ostia in the 4th-3rd c. BC varied between 3.5 m and 4.5 m below the estimated contemporary sea-level, leading to the suggestion that this water space could have served as a “naturally sheltered place on the coast”. Further research in the area may prove that there existed a navigable access to the lagoon at that time, which would strengthen this hypothesis. In the same way, a coastal area that was open to the sea, like offshore Ostia, could also be used as a harbour [32]. PADM charts constructed from the evidence of cores drilled on the coast, in lagoons, channels and lakes can give us an idea of the depth of water columns and their degree of closure. In the same way, the lowest sandy layers of the cores PO-2 and TR-XIV from Ostia and Portus, which dated to the first part of the 1^st^ millennium BC, formed part of a navigable coast with swells close to a river mouth where a ship could moor.

## Conclusion

This paper proposes a comparative synthesis of the harbour basin of Ostia and the pool of Portus using a new chart called *Palaeoenvironmental Age-Depth Model* (PADM). It suggests that there was a high degree of control of the sedimentation by dredging in the harbour of Ostia between the 4^th^ c. BC and the 1^st^ c. AD. The pool of Portus shows similar control of the sedimentation but from the 1^st^ c. AD and the 7^th^ c. AD and probably later. The last deep dredging of the pool (6-7m under the ancient sea-levels) was undertaken in the 3^rd^–4^th^ c. AD. Later dredging horizons seem to affect smaller water columns. Finally, we can observe a very marked change in the 1^st^ c. AD, with the definitive end of the harbour basin of Ostia and the foundation of Portus.

The PADM proposed in this paper provides a new way to visualize integrated data, such as stratigraphic sequences, sea-level indicators, and sedimentation rates reconstructed by using different dating methods, and make it easier to develop comparative interpretations. Applied specifically to the geoarchaeology of harbours, this chart makes it possible to foster a useful transdisciplinary dialogue between geoarchaeologists, archaeologists and historians, that results in more robust interpretations of the navigability and accessibility of ancient water bodies. Fundamentally, therefore, this chart promotes the concept of “harbour potential” in geoarchaeology, whereby each stratigraphic sequence can be studied in terms of the degree to which an ancient water body was closed, the depth of the water column within it, and its suitability for ships of different draughts at different points in time.

Consequently the chart makes it possible to develop comparative studies of enclosed artificial harbours, lagoons, bays, rivers and canals, in order to gauge their potential for shipping at different points in time. Future work will (1) undertake further similar analyses at other Mediterranean ports in order to characterize harbours in terms of navigability, accessibility, harbour potential and harbour operation, and (2) to combine these analyses of harbour depths with new data for the extent of ancient harbour basins. The aim will be to gain a clearer idea of the scale of harbour operation at different Roman ports across the Mediterranean.

## Supporting Information

S1 Fig PADM charts of the cores OST-3 and OST-8 –Harbour of Ostia. (TIF)

S2 Fig PADM charts of the cores CT-1 (*Canale Traverso*) and TR-20 (Entrance to the Trajanic harbour)–Pool of Portus. (TIF)

S3 Fig PADM charts of the cores TR-11 (Claudian harbour) and TR-19 (Pool of Portus). (TIF)

S1 Table Radiocarbon, and archaeological dates. *Calibrated using the Marine13 curve.
(DOC)
