# Correction: The Development and Characteristics of Ancient Harbours—Applying the PADM Chart to the Case Studies of Ostia and Portus

**DOI:** 10.1371/journal.pone.0170140

**Published:** 2017-01-09

**Authors:** Ferreol Salomon, Simon Keay, Nicolas Carayon, Jean-Philippe Goiran

Fig 8 is incorrectly replaced with Fig. 5. Please view the correct Fig 8 here.

**Fig 8:**
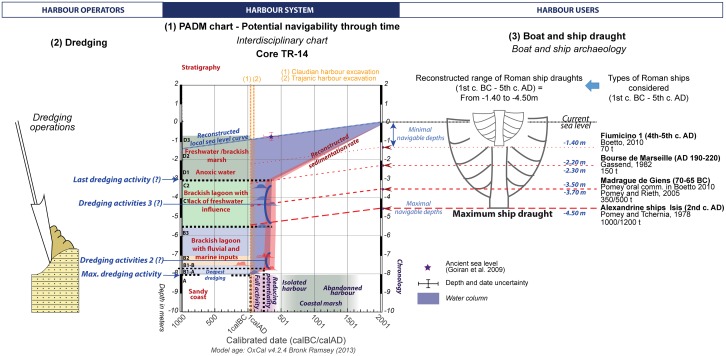
Harbour operation and PADM of the core TR-14. This figure shows the PADM chart of the operating life of the pool of the harbours at Portus as reflected in the sedimentary sequence of core TR-XIV, and expressed by four stratigraphic units. The PADM chart incorporates stratigraphic data as well as factoring in the dredging level hypothesis and possible ship draughts. Deep dredging activities may have occurred in the 3rd -4th c. AD.
