# Supplementary figures and images for: The Development and Characteristics of Ancient Harbours—Applying the PADM Chart to the Case Studies of Ostia and Portus
Source: PLoS One. 2016 Sep 15;11(9):e0162587. doi: 10.1371/journal.pone.0162587 (PMC5025247; doi:10.1371/journal.pone.0162587)

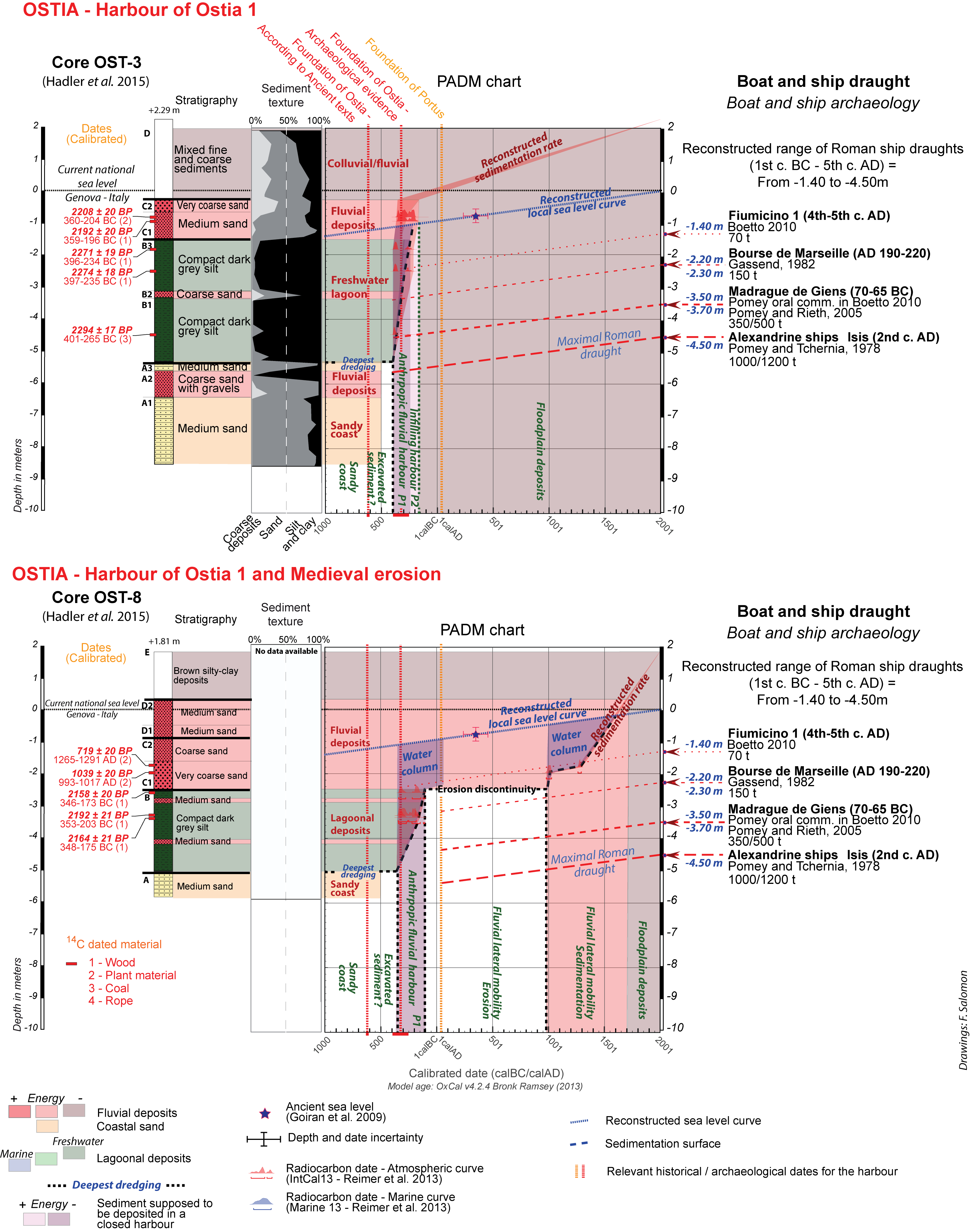

Supplement: S1 Fig — (TIF) [file pone.0162587.s001.tif]

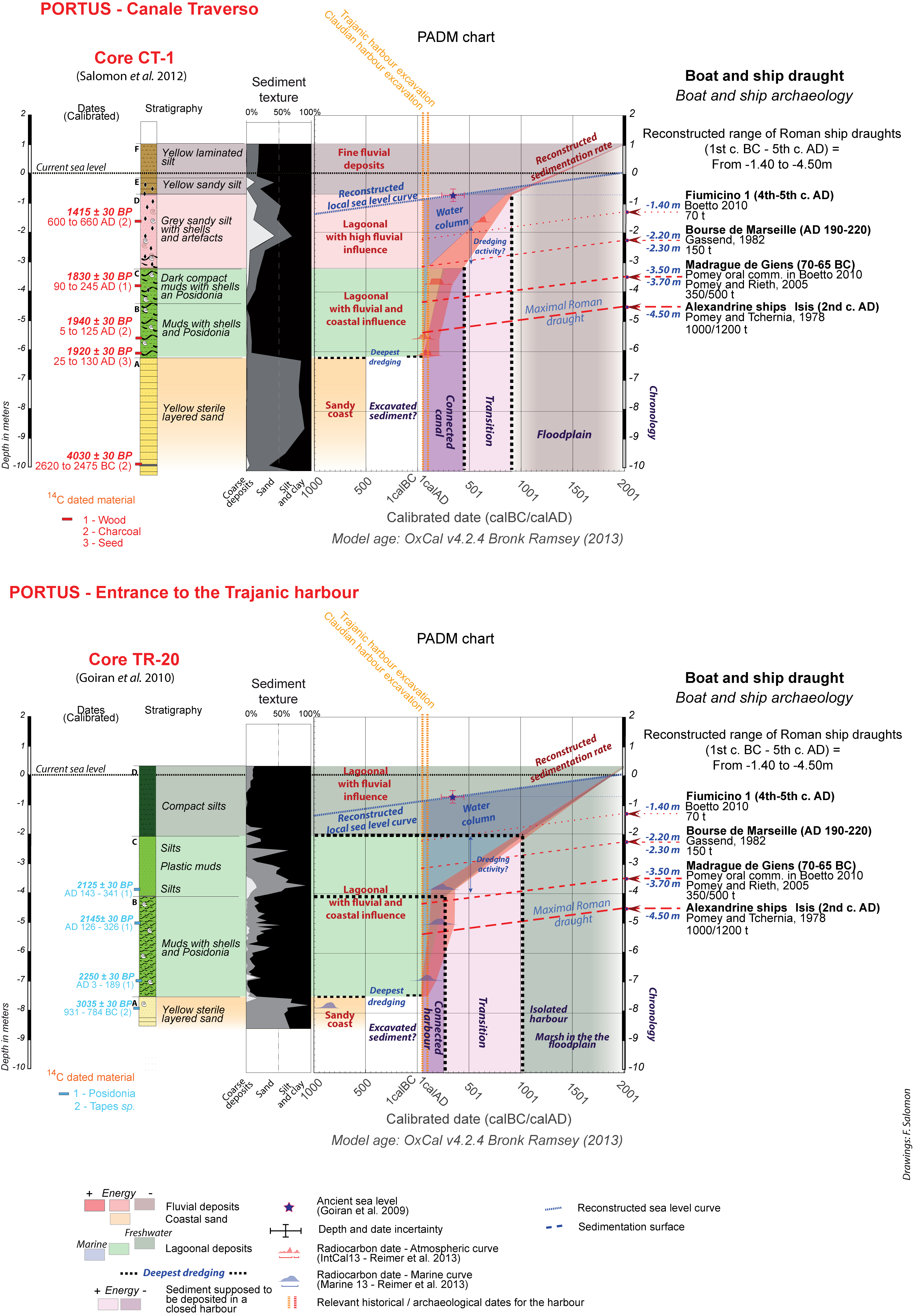

Supplement: S2 Fig — (TIF) [file pone.0162587.s002.tif]

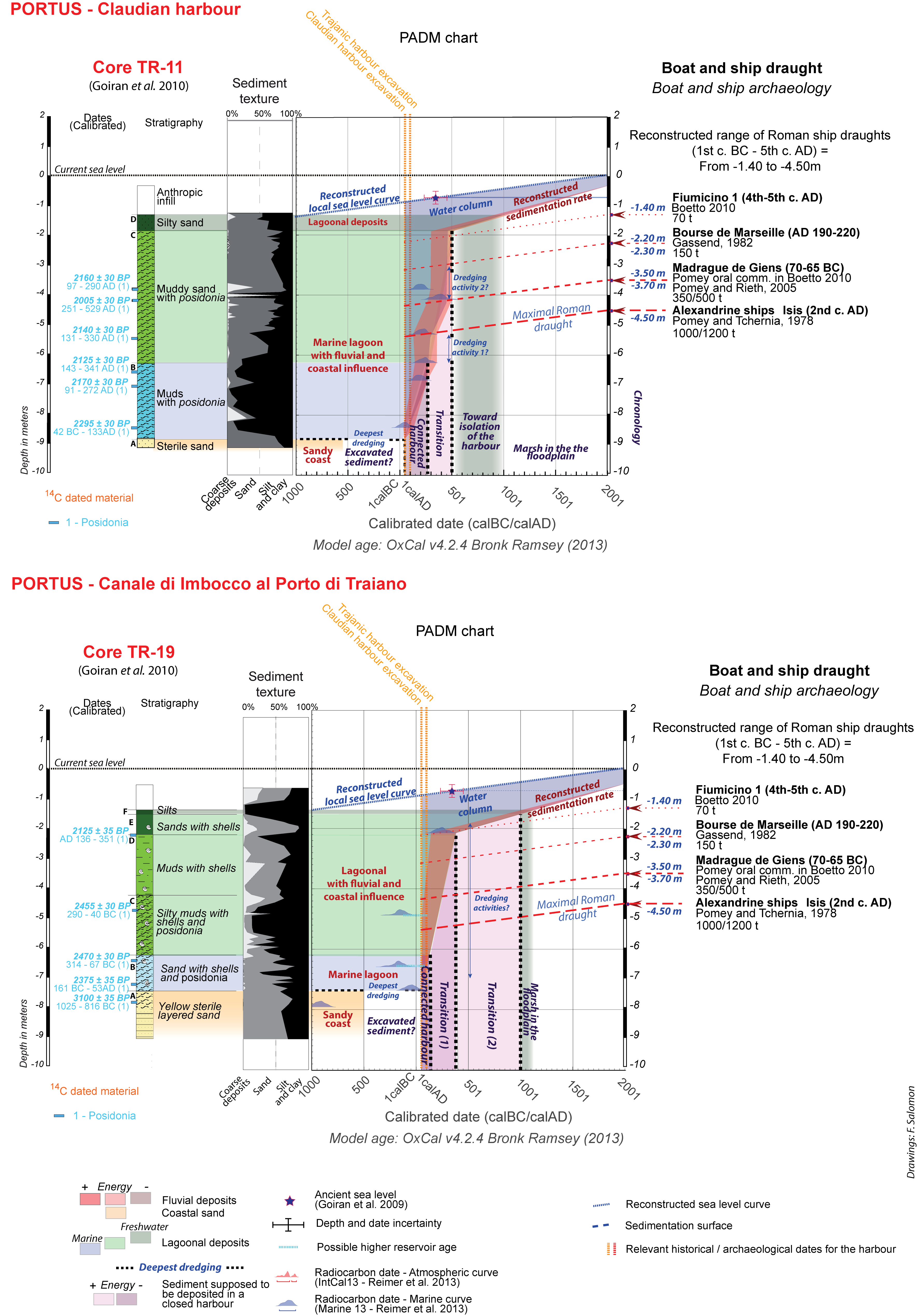

Supplement: S3 Fig — (TIF) [file pone.0162587.s003.tif]
