# Supplementary material for: The Development and Characteristics of Ancient Harbours—Applying the PADM Chart to the Case Studies of Ostia and Portus
Source: PLoS One. 2016 Sep 15;11(9):e0162587. doi: 10.1371/journal.pone.0162587 (PMC5025247; doi:10.1371/journal.pone.0162587)
Supplement: S1 Table — *Calibrated using the Marine13 curve. (DOC) [file pone.0162587.s004.doc]

| **HARBOUR OF OSTIA** | | | | | | | | | |
| --- | --- | --- | --- | --- | --- | --- | --- | --- | --- |
| **Core** | **Samples** | **Depth below surface** | **Depth below sea level (s.l.m - Genoa)** | **Laboratory samples** | **Dating support** | **Radiocarbon dates:**  **14C yr B.P.** | **±** | **Age calibrated BC-AD**  **(Reimer et al., 2013) - 2σ** | **Ref.** |
| PO-2  (+2.50m) | NA | -3.78 | -1.38 | Ly-8059 (GrA) | Wood | 2040 | 25 | 160 BC to AD 25 | Goiran *et al.,* 2014 |
| PO-2 | NA | -4 | -1.6 | Ly-8060 (GrA) | Wood | 2040 | 25 | 160 BC to AD 25 | Goiran *et al.,* 2014 |
| PO-2 | NA | -4.9 | -2.5 | Ly-8061 (GrA) | Charcoal | 1990 | 25 | 44BC to 63AD | Goiran *et al.,* 2014 |
| PO-2 | NA | -4.9 | -2.5 | Ly-8062 (GrA) | Organic matter | 2050 | 25 | 164 BC to 16 AD | Goiran *et al.,* 2014 |
| PO-2 | NA | -5.26 | -2.86 | Ly-8063 (GrA) | Plant material | 2025 | 25 | 98 BC to AD 52 | Goiran *et al.,* 2014 |
| PO-2 | NA | -5.26 | -2.86 | Ly-8064 (GrA) | Wood | 2050 | 25 | 164 BC to AD 16 | Goiran *et al.,* 2014 |
| PO-2 | NA | -6.045 | -3.645 | Ly-9096 (GrA) | Wood | 2160 | 30 | 358 to 108 BC | Goiran *et al.,* 2014 |
| PO-2 | NA | -7.035 | -4.635 | Ly-9095 (GrA) | Charcoal | 2185 | 30 | 361 to 172 BC | Goiran *et al.,* 2014 |
| PO-2 | NA | -8.15 | -5.75 | Ly-9094 (GrA) | Charcoal | 2350 | 40 | 729 to 361 BC | Goiran *et al.,* 2014 |
| PO-2 | NA | -8.53 | -6.13 | Ly-9093 (GrA) | Charcoal | 2125 | 30 | 348 to 52 BC | Goiran *et al.,* 2014 |
| PO-2 | NA | -8.705 | -6.305 | Ly-9092 (GrA) | Wood | 2165 | 30 | 359 to 112 BC | Goiran *et al.,* 2014 |
| PO-2 | NA | -10.5 | -8.1 | Ly-8066 (GrA) | *Posidonia** | 2955 | 25 | 836 to 736 BC | Goiran *et al.,* 2014 |
| PO-1  (+2.36m) | NA | -5.3 | -2.9 | Ly-8045 (GrA) | Wood | 2295 | 30 | 406 to 231 BC | Goiran *et al.,* 2014 |
| PO-1 | NA | -5.73 | -3.35 | Ly-8046 (GrA) | Wood | 2055 | 25 | 165 BC to AD 4 | Goiran *et al.,* 2014 |
| PO-1 | NA | -10.85 | -8.45 | Ly-8047 (GrA) | Plant material | 2670 | 30 | 895 to 798 BC | Goiran *et al.,* 2014 |
| OST-1  (+1.81m) | OST 1/22 HR | -3.8 | -1.99 | MAMS-19743 | Wood | 2193 | 18 | 359 to 197 BC | Hadler *et al.,* 2015 |
| OST-1 | OST 1/26 HR | *-4.64 or ‑4.19* | *-2.38 or ‑2.83* | MAMS-19744 | Wood | 2258 | 18 | 393 to 230 BC | Hadler *et al.,* 2015 |
| OST-1 | OST 1/41 HR | -7.89 | -6.08 | MAMS-19745 | Wood | 43680 | 710 | 46784 to 43629 BC | Hadler *et al.,* 2015 |
| OST-1 | OST 1/49 HR | -10.62 | -8.81 | MAMS-19746 | unident. plant remain | 2628 | 21 | 825 to 791 BC | Hadler *et al.,* 2015 |
| OST-2  (+1.85m) | OST 2/14 PR | -3.34 | -1.49 | MAMS-19747 | unident. plant remain | 1026 | 19 | AD 986 to 1027 | Hadler *et al.,* 2015 |
| OST-3  (+2.92m) | OST 3/14 + HK | -3.7 | -0.78 | MAMS-19748 | Charcoal | 2208 | 20 | 361 to 203 BC | Hadler *et al.,* 2015 |
| OST-3 | OST 3/15 HR | -3.83 | -0.91 | MAMS-19749 | Wood | 2192 | 20 | 360 to 196 BC | Hadler *et al.,* 2015 |
| OST-3 | OST 3/18 + HR | -4.7 | -1.78 | MAMS-19750 | Wood | 2271 | 19 | 397 to 234 BC | Hadler *et al.,* 2015 |
| OST-3 | OST 3/20 + HR | -5.38 | -2.46 | MAMS-19751 | Wood | 2274 | 18 | 398 to 235 BC | Hadler *et al.,* 2015 |
| OST-3 | OST 3/28 PR | -7.35 | -4.43 | MAMS-19752 | piece of rope | 2294 | 17 | 401 to 265 BC | Hadler *et al.,* 2015 |
| OST-4  (+4.38m) | OST 4/14 HK | -5.72 | -1.34 | MAMS-19753 | Charcoal | 2229 | 17 | 376 to 207 BC | Hadler *et al.,* 2015 |
| OST-4 | OST 4/19 + PR | -6.68 | -2.30 | MAMS-19754 | unident. plant remain | 2562 | 19 | 802 to 597 BC | Hadler *et al.,* 2015 |
| OST-5  (+2.56m) | OST 5/4 HK | -1.74 | 0.82 | MAMS-19755 | Charcoal | 1999 | 19 | 44 BC to AD 53 | Hadler *et al.,* 2015 |
| OST-5 | OST 5/6 HK | -2.34 | 0.22 | MAMS-19756 | Charcoal | 1930 | 18 | AD 26 to 125 | Hadler *et al.,* 2015 |
| OST-5 | OST 5/8 HK | -2.78 | -0.22 | MAMS-19757 | Charcoal | 2110 | 20 | 196 to 56 BC | Hadler *et al.,* 2015 |
| OST-5 | OST 5/19 HR | -4.36 | -1.80 | MAMS-19758 | Wood | 2149 | 20 | 351 to 111 BC | Hadler *et al.,* 2015 |
| OST-5 | OST 5/22 PR | -4.65 | -2.09 | MAMS-19759 | unident. plant remain | 2255 | 20 | 393 to 311 BC | Hadler *et al.,* 2015 |
| OST-6  (+2.04m) | OST 6/13 HR | -3.88 | -1.84 | MAMS-19760 | Wood | 2101 | 20 | 183 to 52 BC | Hadler *et al.,* 2015 |
| OST-6 | OST 6/19 + HR | -4.56 | -2.52 | MAMS-19761 | Wood | 2168 | 20 | 356 to 166 BC | Hadler *et al.,* 2015 |
| OST-6 | OST 6/27 + PR | -7.42 | -5.38 | MAMS-19762 | unident. plant remain | 2191 | 20 | 360 to 395 BC | Hadler *et al.,* 2015 |
| OST-8  (+1.08m) | OST 8/15 PR | -2.82 | -1.74 | MAMS-19763 | unident. plant remain | 719 | 20 | AD 1263 to 1293 | Hadler *et al.,* 2015 |
| OST-8 | OST 8/16 PR | -3 | -1.92 | MAMS-19764 | unident. plant remain | 1039 | 20 | AD 977 to 1025 | Hadler *et al.,* 2015 |
| OST-8 | OST 8/23 HR | -3.62 | -2.54 | MAMS-19765 | Wood | 2158 | 20 | 355 to 116 BC | Hadler *et al.,* 2015 |
| OST-8 | OST 8/28 HR | -4.33 | -3.25 | MAMS-19766 | Wood | 2192 | 21 | 360 to 195 BC | Hadler *et al.,* 2015 |
| OST-8 | OST 8/29 HR | -4.39 | -3.31 | MAMS-19767 | Wood | 2164 | 21 | 356 to 119 BC | Hadler *et al.,* 2015 |
| **Core** | **Sample** | **Depth bellow surface** | **Depth below sea level (s.l.m - Genoa)** | **Sample description** | | | | **Age estimation** | **Ref.** |
| OST-1  (+1.81m) | OST 1/-K | -3.60 | -1.75 | Fragment of moulded lamp, Augustan period | | | | 27 BC – AD 14 or younger | Hadler *et al.*, 2015 |
| OST-3  (+2.92m) | OST 3/9 þ K | *-3.11 or -3.74* | *-0.82 or ‑0.19* | Body sherd of Italian sigillata | | | | 440 BC – AD 150 | Hadler *et al.*, 2015 |
| OST-3 | OST 3/11 K | *-3.36 or -3.99* | *-1.07 or  ‑0.44* | Body sherd of Italian sigillata | | | | 40 BC – AD 150 | Hadler *et al.*, 2015 |
| OST-5  (+2.56m) | OST 5/4 K | -1.70 | 0.86 | Body sherd of Italian sigillata | | | | 40 BC – AD 150 | Hadler *et al.*, 2015 |
| OST-5 | OST 5/7 K | -2.53 | 0.03 | Italian sigillata, fragment from edge of plate (Conspectus No. 20.3), late Augustan to Tiberian period | | | | AD 1 – 30 | Hadler *et al.*, 2015 |
| **HARBOUR POOL OF PORTUS** | | | | | | | | | |
| **Core** | **Samples** | **Depth bellow surface** | **Depth below sea level (s.l.m - Genoa)** | **Laboratory samples** | **Dating support** | **Radiocarbon dating:**  **14C yr B.P.** | **±** | **Age calibrated BC-AD**  **(Reimer et al., 2013) - 2**σ | **Ref.** |
| CT-1  (+1.80m) | CT1-B (1158) | -11.58 | -9.78 | Lyon-6870 | Charcoal | 4030 | 30 | 2623 to 2473 BC | Salomon *et al.*, 2012 |
| CT-1 | CT 1-G (792) | -7.92 | -6.12 | Lyon-6895 | Seed | 1920 | 30 | AD 3 to 204 | Salomon *et al.*, 2012 |
| CT-1 | CT 1-E (737) | -7.37 | -5.57 | Lyon-6894 | Charcoal | 1940 | 30 | 20 BC to AD 130 | Salomon *et al.*, 2012 |
| CT-1 | CT1-C (554) | -5.53 | -3.74 | Lyon-7081 | Wood | 1830 | 30 | AD 86 to 311 | Salomon *et al.*, 2012 |
| CT-1 | CT1-A (344) | -3.44 | -1.64 | Lyon-6869 | Charcoal | 1415 | 30 | AD 585 to 663 | Salomon *et al.*, 2012 |
| TR-XI  (-0.37m) | S 11-25 | -3.59 | -3.96 | Poz-17594 | *Posidonia** | 2160 | 30 | AD 97 to 290 | Goiran *et al.,* 2010 |
| TR-XI | S 11 31 | -3.895 | -4.27 | Poz-16100 | *Posidonia** | 2005 | 50 | AD 251 to 529 | Goiran *et al.,* 2010 |
| TR-XI | S 11-53 | -5.075 | -5.45 | Poz-17595 | *Posidonia** | 2140 | 30 | AD 131 to 330 | Goiran *et al.,* 2010 |
| TR-XI | S 11-61 | -6.05 | -6.42 | Ly-4042 | *Posidonia** | 2125 | 30 | AD 143 to 341 | Goiran *et al.,* 2010 |
| TR-XI | S 11-67 | -6.65 | -7.02 | Ly-4044 | *Posidonia** | 2170 | 30 | AD 91 to 272 | Goiran *et al.,* 2010 |
| TR-XI | S 11-81 | -8.25 | -8.62 | Poz-17596 | *Posidonia** | 2295 | 30 | 42 BC to AD 133 | Goiran *et al.,* 2010 |
| TR-XIV  (-0.13m) | d8b | -3.43 | -3.56 | Lyon-7474 | *Vegetal matter** | 2160 | 30 | AD 97 to 290 | Delile *et al.,* 2014 |
| TR-XIV | d8a | -3.43 | -3.56 | Lyon-8067 | *Posidonia** | 2165 | 25 | AD 102 to 269 | Delile *et al.,* 2014 |
| TR-XIV | d7 or TR14 403 - 406 | -4.05 | -4.18 | Lyon-8068 (SacA 24067) | *Posidonia** | 2145 | 25 | AD 131 to 315 | Delile *et al.,* 2014 |
| TR-XIV | d6 | -4.34 | -4.47 | Lyon-8069 | *Posidonia** | 2035 | 25 | AD 265 to 423 | Delile *et al.,* 2014 |
| TR-XIV | d5 | -5.49 | -5.62 | Lyon-7470 | *Vegetal matter** | 2140 | 30 | AD 131 to 330 | Delile *et al.,* 2014 |
| TR-XIV | d4 | -6.98 | -7.11 | UCIAMS-114467 | Wood | 1790 | 20 | AD 138 to 325 | Delile *et al.,* 2014 |
| TR-XIV | d3 | -7.04 | -7.17 | Lyon-8777 | Wood | 1765 | 30 | AD 142 to 379 | Delile *et al.,* 2014 |
| TR-XIV | d2b | -7.69 | -7.82 | Lyon-8876 | Wood | 1710 | 35 | AD 246 to 401 | Delile *et al.,* 2014 |
| TR-XIV | d2a | -7.69 | -7.82 | Lyon-8877 | Charcoal | 2080 | 25 | 176 to 41 BC | Delile *et al.,* 2014 |
| TR-XIV | d1 | -7.90 | -8.03 | Lyon-8776 | *Posidonia** | 2250 | 30 | AD 3 to 189 | Delile *et al.,* 2014 |
| TR-XIX  (-0.49m) | TR XIX 13 | -1.63 | -2.12 | Poz-16101 | *Posidonia** | 2125 | 35 | AD 136 to 351 | Goiran *et al.,* 2010 |
| TR-XIX | TR XIX 27 | -4.36 | -4.85 | Poz-16234 | *Posidonia** | 2455 | 30 | 290 to 40 BC | Goiran *et al.,* 2010 |
| TR-XIX | TR XIX 46 | -6.065 | -6.56 | Poz-16104 | *Posidonia** | 2470 | 30 | 314 to 67 BC | Goiran *et al.,* 2010 |
| TR-XIX | TR XIX 59 posi | -6.86 | -7.35 | Ly-4244 | *Posidonia** | 2375 | 35 | 161 BC to AD 53 | Goiran *et al.,* 2010 |
| TR-XIX | TR XIX 62 | -7.4 | -7.89 | Poz-16280 | *Posidonia** | 3100 | 35 | 1025 to 816 BC | Goiran *et al.,* 2010 |
| TR-XX  (+0.33m) | TR XX 83 | -4.175 | -3.85 | Ly-4041 (SacA 6585) | *Posidonia** | 2125 | 30 | AD 143 to 341 | Goiran *et al.,* 2010 |
| TR-XX | TR XX 109 | -5.35 | -5.02 | Ly-4043 (SacA 6587) | *Posidonia** | 2145 | 30 | AD 126 to 326 | Goiran *et al.,* 2010 |
| TR-XX | TR XX 153 posi | -7.235 | -6.91 | Ly-4045 (SacA 6589) | *Posidonia** | 2250 | 30 | AD 3 to 189 | Goiran *et al.,* 2010 |
| TR-XX | TR XX 173 | -8.16 | -7.83 | Ly-4035 (SacA 6576) | *Shell - Tapes* sp.*** | 3035 | 30 | 931 to 784 BC | Goiran *et al.,* 2010 |

Supplementary material A4 – **Radiocarbon, and archaeological dates.**Calibrated using the Marine13 curve***
